# Nationwide Analysis of Legal Barriers to Cancer Care

**DOI:** 10.1001/jamanetworkopen.2025.24201

**Published:** 2025-07-31

**Authors:** Krista Y. Chen, Amanda L. Blackford, Monica F. Bryant, Joanna F. Doran, Nicole L. Henderson, Andres Azuero, Gabrielle B. Rocque, S. M. Qasim Hussaini

**Affiliations:** 1 School of Medicine, Johns Hopkins University School of Medicine, Baltimore, Maryland; 2 Division of Quantitative Sciences, Sidney Kimmel Comprehensive Cancer Center, Johns Hopkins University School of Medicine, Baltimore, Maryland; 3 Triage Cancer, Chicago, Illinois; 4 Department of Medicine, O’Neal Comprehensive Cancer Center, University of Alabama at Birmingham; 5 School of Nursing, University of Alabama at Birmingham

## Abstract

**Importance:**

Legal barriers during cancer care contribute to longstanding disparities and lead to adverse health outcomes in vulnerable populations, yet their prevalence remains unknown.

**Objective:**

To identify and characterize legal barriers for patients with cancer who reached out to a legal navigation program.

**Design, Setting, and Participants:**

This retrospective cohort study used data from the Legal & Financial Navigation (LFN) database of Triage Cancer, a nationwide nonprofit organization providing free education on legal issues faced by individuals with cancer, between March 1, 2021, and December 31, 2024. Participants included patients with cancer and caregivers and health care professionals calling on a patient’s behalf.

**Exposures:**

Sociodemographic, financial, and disease-site characteristics of patients.

**Main Outcomes and Measures:**

The main outcome was the primary legal issue prompting use of Triage Cancer’s LFN. Sociodemographic, financial, and disease-site factors of patients were characterized, and the associations of these factors with the primary legal issue were explored with logistic regression models.

**Results:**

A total of 5810 calls (3883 [66.8%] by patients, 1091 [18.8%] by caregivers, and 597 [10.3%] by health care professionals) were investigated in reference to patients from 50 US states (3710 [63.9%] female; 3293 [56.7%] aged 40 to 64 years). Patients were primarily insured (4436 [76.4%]), 2533 (43.6%) had annual household income below $50 000, and 2808 (48.3%) were in active cancer treatment. Almost half of patients (2807 [48.3%]) had 2 or more legal barriers. The most common primary barriers were health insurance (1648 [28.4%] requested help navigating claim denials, appeals, or health insurance), followed by finances (1194 [20.6%] needed help with financial assistance and housing), employment (1095 [18.8%] needed help navigating wrongful termination, working through treatment, taking time off, or unemployment benefits), and disability insurance (1082 [18.6%] needed help navigating claim denials or applying for and transitioning off disability insurance). Black or African American compared with White patients had lower odds of needing assistance with health insurance (odds ratio [OR], 0.66; 95% CI, 0.50-0.87); Black or African American compared with White patients (OR, 1.52; 95% CI, 1.12-2.05) and patients living in the South compared with the Northeast (OR, 1.32; 95% CI, 1.04-1.67) more often sought assistance for financial barriers; and patients with household income below $20 000 compared with over $100 000 (OR, 0.34; 95% CI, 0.14-0.87) and those with Medicaid (OR, 0.36; 95% CI, 0.13-0.99) or marketplace insurance (OR, 0.37; 95% CI, 0.23-0.60) vs employer-sponsored insurance less often sought assistance for employment.

**Conclusions and Relevance:**

This cohort study examining legal barriers to cancer care access faced by patients with cancer and their caregivers found that certain populations may have greater need for legal navigation. In light of recent federal policies supporting reimbursement for complex care coordination and unmet social services needs in patients with cancer, these findings suggest a need for health care teams to better understand cancer-related legal issues and design more accessible legal navigation services.

## Key Points

**Question:**

What are the predominant legal challenges faced by individuals diagnosed with cancer who seek legal navigation?

**Findings:**

In this cohort study of 5810 patients, caregivers, and health care professionals using legal navigation services, 48% of patients faced 2 or more legal barriers to health insurance, financial assistance, disability benefits, employment, and more. Black or African American and low-income populations made more requests for assistance with finances, while White and high-income populations more frequently sought assistance with health insurance and employment.

**Meaning:**

The findings suggest accessible legal navigation services and education for health care professionals are needed to address legal barriers during cancer treatment and beyond.

## Teaser

This cohort study characterizes legal barriers to cancer care access for which patients with cancer use legal navigation services and identifies factors associated with greater need for specific areas of legal intervention.

## Introduction

Individuals diagnosed with cancer often face challenges that extend far beyond their medical treatment, impacting care across diverse populations.^1,2,3,4,5^ In recent years, cancer care has become increasingly complex and costly—marked by multistep evaluations including imaging, genetic testing, and biopsies; multimodal treatments integrating medical, radiation, and surgical oncology teams; novel, high-cost pharmaceuticals; and increased patient cost-sharing.^6,7,8,9^ Given this, timely coordination and navigation are critical to reducing barriers and addressing widening disparities in outcomes.^10,11^ While social determinants of health, such as income, educational level, and housing, are well-recognized contributors to these disparities, less emphasis has been placed on the legal structures that also play an integral role in shaping access to care and exacerbating cancer disparities.^12,13^ Otherwise known as legal determinants of health, these factors encompass the laws, regulations, and policies that govern eligibility for health care, employment protections, and other essential services and benefits.^14^ While the law can function to enable access to health services, unequal burden of legal barriers can delay or prevent access to consistent, quality, and comprehensive cancer care.^15^

Social workers, community health workers, and patient navigators have long played a vital role in helping patients navigate the intersection of legal, financial, and health care systems.^16,17,18^ However, the regulatory landscape has become increasingly complex, and the solutions to barriers faced by patients may be entrenched in local, state, and federal policies. Legal experts may be specifically positioned to alleviate some of the emotional burden and financial strain imposed by cancer care and emerge as a vital, underused resource.^19,20^ Medical-legal partnerships are 1 solution that has emerged in response to this growing need, embedding lawyers in health care settings such as outpatient medical clinics to assist patients from underresourced communities with legal needs as they arise.^21^ However, these partnerships are often tied to institutions and are thereby geographically limited; only a handful of small-cohort studies have assessed prevalence of medically related legal needs at the institutional level.^22,23^ To our knowledge, no study to date has investigated health-related legal barriers at a national level or in a diverse cancer population. Furthermore, whether patients of certain sociodemographic groups or with certain types of cancer are more vulnerable to specific legal needs remains unclear.

Our study aimed to fill this gap by investigating legal barriers to cancer care access requiring legal navigation in patients with cancer. In this retrospective study, we included, to our knowledge, the first nationwide cohort of patients with cancer receiving legal navigation services. We characterized the sets of challenges faced by this population and assessed sociodemographic, financial, and disease-site factors associated with greater need for specific areas of legal intervention.

## Methods

### Study Setting and Cohort

This was a retrospective cohort study of individuals who consulted Triage Cancer’s Legal & Financial Navigation Program (LFN) between March 1, 2021, and December 31, 2024. Triage Cancer is a national nonprofit organization that provides free education on legal and practical issues impacting individuals diagnosed with cancer. It provides one-on-one assistance in areas such as health and disability insurance, finances, employment, and estate planning and medical decision-making for individuals with cancer and for caregivers and health care professionals calling on a patient’s behalf. The University of Alabama at Birmingham considered the present study exempt from institutional review board approval and informed consent due to the use of deidentified data from the LFN electronic database, and it followed the Strengthening the Reporting of Observational Studies in Epidemiology (STROBE) reporting guideline for cohort studies.

Callers completed an online survey to request consultation, providing the patient’s demographic information, socioeconomic status, and cancer characteristics. They were then invited to schedule a consultation within a week of the request. A Triage Cancer staff attorney then conducted a one-on-one telephone call to address legal barriers and navigate individuals to relevant resources and services. If the request was flagged for more urgent issues, staff may have reached out sooner. The intake survey was available in English or Spanish, and Spanish-speaking staff attorneys conducted calls in Spanish when needed. Interpretation services were also available for other languages. Surveys and consultations were designed and conducted by Triage Cancer staff attorneys.

### Covariates and Outcomes

Individual-level measures included patient’s age at consultation; self-reported gender, race, and ethnicity; language; state of residence; and the caller’s relationship to the patient (eg, if the caller identified as a caregiver or health care professional). Race and ethnicity were included because of extensive literature showing their association with different cancer outcomes; categories were American Indian or Alaska Native; Asian or Pacific Islander; Black or African American; Hispanic or Latinx; Middle Eastern or North African; White; other race, ethnicity, or origin (a selectable option on the intake survey); and unknown race or ethnicity or preferred not to share. Information on primary and secondary health insurance, employment status, household income, household size, cancer type, and treatment stage (eg, pretreatment, in treatment, or posttreatment) was also collected from self-reported survey data. Employment status was collected as a free-text response, where individuals reported “job/occupation”; all responses were reviewed and categorized into groups including “employed,” “unemployed—looking for work,” “unemployed—unable to work,” “retired,” “on leave,” or “other” (included children, students, stay-at-home parents, and more) after data collection was complete.

The study’s main outcome was the primary legal issue prompting a call to Triage Cancer. During consultations, callers and legal consultants discussed specific issues and categorized concerns from a list of 28 common legal barriers to care access predetermined by Triage Cancer staff (eTable 1 in Supplement 1). For individuals requiring assistance with multiple barriers, these were ranked in order of priority by the caller and consultant, considering time spent discussing each one. Afterward, legal consultants recorded this information in the deidentified database. During analysis, the research team in collaboration with Triage Cancer staff further grouped the issues into 5 main categories: health insurance (including Medicaid, Medicare, understanding insurance options, navigation, and claim denial appeals), finances (housing; financial assistance, including grants; benefits applications; charity care; and copayment assistance), employment (caregiver rights, getting fired, taking time off, working through treatment, returning to work, job search, and unemployment benefits), disability insurance (applying for, transitioning off, and claim denial appeals), and other issues (COVID-19; education; estate planning, including wills and advanced directives; family law; immigration; and life insurance) (eTable 1 in Supplement 1).

### Statistical Analysis

Descriptive analyses were conducted to characterize the sociodemographic, financial, and disease-site composition of the entire cohort and as stratified by the primary legal issue (the issue for which the greatest amount of time was spent during each call). The main analyses explored associations between patient characteristics and each primary legal issue to assess factors associated with 1 navigation need in comparison with all other legal navigation needs (eg, we explored factors predisposing calls for a primary issue of health insurance as opposed to a non–health insurance issue). For each primary issue, the outcome was defined as a binary variable, specified as having the primary issue (“yes”) vs having 1 of the other 4 issues (“no”). A total of 4 primary-legal-issue outcomes were explored: health insurance, financial, disability insurance, and employment.

Four sets of analyses were chosen in favor of a single multinomial outcome to facilitate interpretation of factors associated with each primary legal issue, as there was no logical hierarchy to the issues that would facilitate a clear reference group for comparisons. Eight explanatory variables (type of caller, race and ethnicity, age group, gender, region, type of health insurance, household income, and cancer site) were chosen for analysis based on purported influence of these variables on sociolegal barriers. Callers with responses including nonbinary, transgender, or other for gender; international or US territory for region; and high risk or seeking preventive health care services for cancer type were removed from the analyses, as sample sizes were too small for analysis and there was no other category into which we could reasonably group them.

Multiple imputation was used to account for missing data across the subset of 8 variables (ie, individuals who omitted a response or chose “prefer not to answer”).^24,25^ This method works by filling in missing values based on observed relationships in the data. Five multiply imputed datasets were generated, and subsequent results were pooled to account for the variation introduced by each imputation. Multivariable models were estimated with logistic regression to describe factors independently associated with having the primary legal issue, and contrasts are reported as odds ratios (ORs) with associated 95% CIs. Model-estimated probabilities for each of the ORs were computed to aid in interpretation.

User ratings of navigation services were tabulated as frequencies and percentages. All statistical analyses were conducted in R, version 4.3.2 (R Project for Statistical Computing).

## Results

### Cohort Characteristics

Overall, 5810 unique calls were made: 3883 (66.8%) by individuals diagnosed with cancer, 1091 (18.8%) by caregivers, and 597 (10.3%) by health care professionals, including social workers (400 [6.9%]), patient advocates and community health workers (99 [1.7%]), nurses (72 [1.2%]), and financial navigators (26 [0.4%]). The calls were by or on behalf of a patient population of 5810 individuals, of whom 17 (0.3%) were American Indian or Alaska Native; 277 (4.8%) were Asian or Pacific Islander; 597 (10.3%) were Black or African American; 598 (10.3%) were Hispanic or Latinx; 51 (0.9%) were Middle Eastern or North African; 2840 (48.9%) were White; 129 (2.2%) were other race, ethnicity, or origin; and 1301 (22.4%) had unknown race or ethnicity or preferred not to share. A total of 3710 patients (63.9%) were female, 1697 (29.2%) were male, 32 (0.6%) were other gender, and 371 (6.4%) preferred not to share; 3293 (56.7%) were aged 40 to 64 years (Table 1). The cohort represented all 50 US states and the District of Columbia, and 82 individuals (1.4%) were from outside the US. The highest percentage of callers were from California (898 [15.5%]), Texas (435 [7.5%]), Illinois (329 [5.7%]), New York (308 [5.3%]), and Florida (304 [5.2%]) (eFigure in Supplement 1).

**Table 1.** Demographic and Disease Site Characteristics of Cohort^a^

Variable	Patients, No. (%) (N = 5810)
Type of caller	
Individual diagnosed with cancer	3883 (66.8)
Caregiver	1091 (18.8)
Social worker	400 (6.9)
Community health worker or patient advocate	99 (1.7)
Nurse	72 (1.2)
Financial navigator	26 (0.4)
Other^b^	229 (3.9)
Unknown	10 (0.2)
Age of patient at time of call, y	
0-39	968 (16.7)
40-64	3293 (56.7)
≥65	807 (13.9)
Unknown or prefer not to share	742 (12.8)
Patient gender	
Female	3710 (63.9)
Male	1697 (29.2)
Other^c^	32 (0.6)
Unknown or prefer not to share	371 (6.4)
Patient race and ethnicity	
American Indian or Alaska Native	17 (0.3)
Asian or Pacific Islander	277 (4.8)
Black or African American	597 (10.3)
Hispanic or Latinx	598 (10.3)
Middle Eastern or North African	51 (0.9)
White	2840 (48.9)
Other race, ethnicity, or origin^d^	129 (2.2)
Unknown or prefer not to share	1301 (22.4)
US Region	
South	1786 (30.7)
West	1482 (25.5)
Midwest	1011 (17.4)
Northeast	997 (17.2)
International	82 (1.4)
US territory	4 (0.1)
Unknown	448 (7.7)
Primary language other than English	167 (3.2)
Primary health insurance	
Employer-sponsored	1977 (34.0)
Medicare	862 (14.8)
Medicaid	745 (12.8)
Individually purchased	585 (10.1)
Uninsured	330 (5.7)
Other, military, or veteran	267 (4.6)
Unknown	1044 (18.0)
Employment status	
Employed	2313 (39.8)
Unemployed—unable to work	518 (8.9)
Unemployed—looking for work	505 (8.7)
Retired	340 (5.9)
Medical leave	33 (0.6)
Other^e^	82 (1.4)
Unknown	1935 (33.3)
Annual household income, $	
<13 000	907 (15.6)
13 000 to <20 000	471 (8.1)
20 000 to <50 000	1155 (19.9)
50 000 to <100 000	993 (17.1)
≥100 000	581 (10.0)
Unknown or prefer not to share	1703 (29.3)
Individuals in household, No.	
1	1648 (28.4)
2	1597 (27.5)
3	668 (11.5)
≥4	778 (13.4)
Unknown	1119 (19.3)
Disease site	
Breast	1618 (27.8)
Hematologic	1134 (19.5)
Gastrointestinal	572 (9.8)
Gynecologic	358 (6.2)
Lung	355 (6.1)
Neuro-oncologic	319 (5.5)
Genitourinary	224 (3.9)
High risk or seeking preventive services	33 (0.6)
Other solid cancer	689 (11.9)
Unknown	508 (8.7)
Stage of treatment	
Pretreatment	350 (6.0)
In treatment	2808 (48.3)
Posttreatment	717 (12.3)
Unknown	1935 (33.3)

^a^ Information was self-reported by participants in a survey prior to consultation. Information was reported for the patient experiencing legal barriers, not the participant serving as their proxy.

^b^ Includes high-risk individuals seeking preventive services and other callers.

^c^ Includes nonbinary nonconforming, transgender, and gender not listed.

^d^ Participants self-reported as “other race, ethnicity, origin.” This category does not include any groupings made by the research team after data collection.

^e^ Includes children, students, stay-at-home parents, and more.

Most patients (4436 [76.4%]) were insured, primarily via employer-sponsored health insurance (1977 [34.0%]), Medicare (862 [14.8%]), Medicaid (745 [12.8%]), or individually purchased health insurance (585 [10.1%]), while 330 (5.7%) were uninsured. Annual household income was well distributed, with 2533 callers (43.6%) reporting patient income below $50 000 (1378 [23.7%] less than $20 000 and 1155 [19.9%] from $20 000 to less than $50 000) and 993 (17.1%) reporting income from $50 000 to less than $100 000. Household size varied from 1648 (28.4%) living alone to 778 (13.4%) living in households with 4 or more individuals. While 2313 patients (39.8%) were employed at the time of the call, 1396 (24.0%) were unemployed, on medical leave, or retired. Anecdotally, many patients reported that their unemployment status—whether from being fired, having to leave work, having to retire early, or an inability to find work—was directly related to their cancer diagnosis and/or treatment.

The proportion of missingness in our data for the 8 variables was 10 (0.2%) for type of caller, 1301 (22.4%) for patient race and ethnicity, 742 (12.8%) for patient age, 371 (6.4%) for patient gender, 448 (7.7%) for US region, 1044 (18.0%) for primary health insurance, 1703 (29.3%) for household income, and 508 (8.7%) for disease site (Table 1). A total of 151 patients, representing 2.6% of the cohort, reported either nonbinary, transgender, or other response for gender; international or US territory for region; or high risk or seeking preventive health care services for cancer type and were removed from the analyses owing to small sample size.

### Disease-Specific Characteristics

Patients had more than 30 different primary cancer types, with the most common being breast (1618 [27.8%]), hematologic (1134 [19.5%]), gastrointestinal (572 [9.8%]), gynecologic (358 [6.2%]), and lung (355 [6.1%]) (Table 1). A total of 2808 patients (48.3%) were in treatment, while 350 (6.0%) were pretreatment and 717 (12.3%) were posttreatment; treatment stage was unknown for 1935 (33.3%).

### Use of Legal Navigation Services

**Figure 1.**
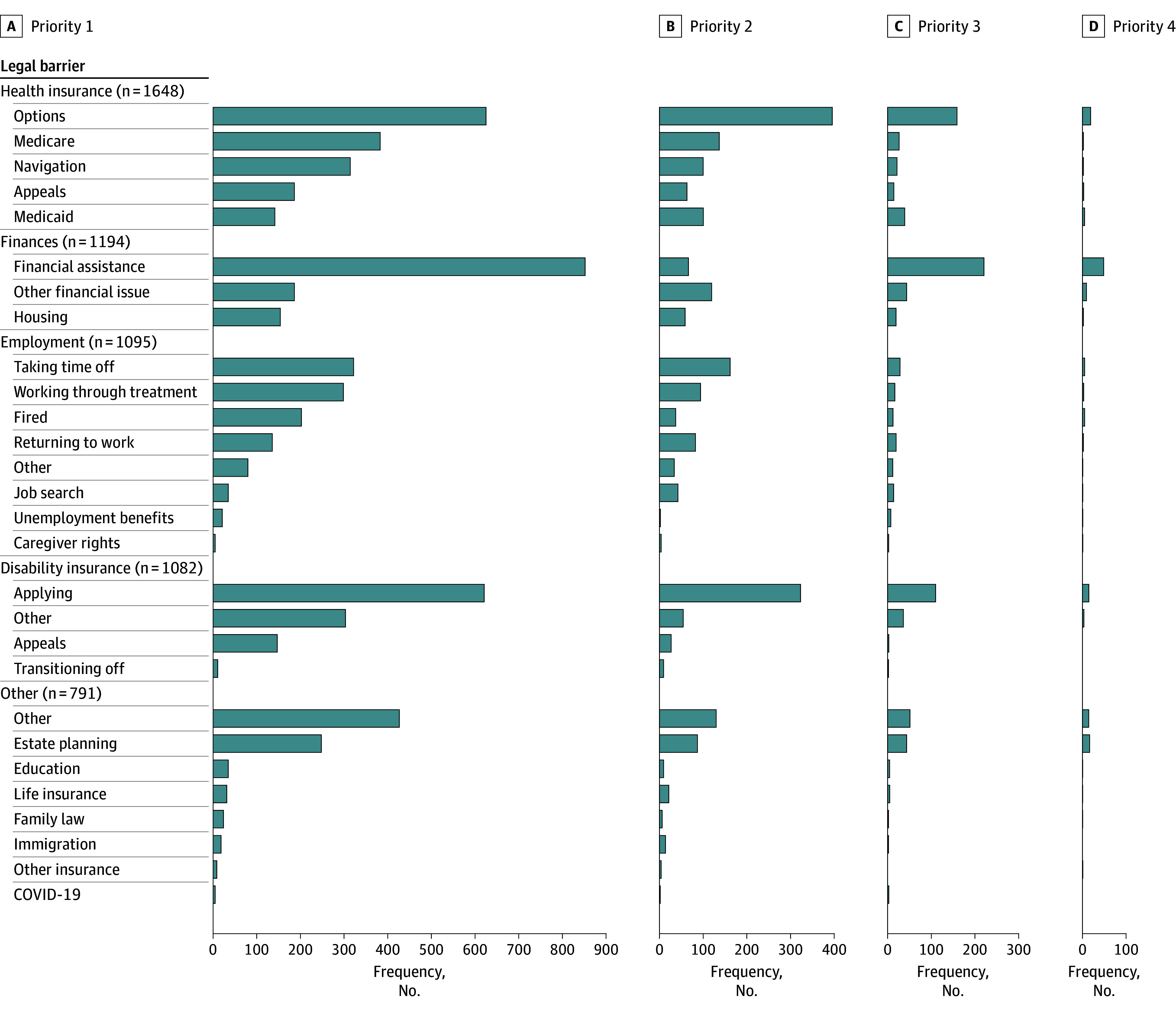
Legal Navigation Needs By Priority Frequencies were organized by priority of each barrier; in the case that an individual required assistance with multiple legal barriers, legal consultants ranked all barriers from highest (first) to lowest priority based on amount of time discussing each legal need. The number of legal barriers per consultation, and thereby priority of legal barriers, was capped at 4.

Across 5810 calls, requests for legal navigation services were made for 9755 instances of barriers, with most patients (3003 [51.7%]) calling for 1 legal issue and 2807 (48.3%) calling for more than 1: 1869 (32.2%) called for 2, 738 (12.7%) for 3, and 200 (3.4%) for 4 (Table 2). The most common primary issue was related to health insurance (options, appeals; 1648 patients [28.4%]), followed by finances (assistance, housing; 1194 [20.6%]), employment (working through treatment, taking time off, wrongful termination; 1095 [18.8%]), and disability insurance (application, appeals; 1082 [18.6%]). Sizable proportions of all 9755 concerns discussed were related to employment (1719 [17.6%]) or disability insurance (1771 [18.2%]). Figure 1 describes the various legal barriers discussed in calls and the frequency of each topic, ranked by priority. All patient characteristics in Table 1 were additionally stratified by primary legal issue for comparison across groups and are represented in eTable 2 in Supplement 1.

**Table 2.** Frequency of Legal Navigation Service Requests

Barrier^a^	Callers, No. (%) (N = 5810)^b^			
	First priority	Second priority	Third priority	Fourth priority
Health insurance	1648 (28.4)	800 (28.1)	266 (28.4)	39 (19.3)
Finances	1194 (20.6)	747 (26.2)	286 (30.5)	67 (33.2)
Employment	1095 (18.8)	472 (16.6)	122 (13.0)	30 (14.9)
Disability insurance	1082 (18.6)	513 (18.0)	155 (16.5)	21 (10.4)
Other	791 (13.6)	273 (9.6)	109 (11.6)	45 (22.3)

^a^ Barriers discussed within a single consultation. The number of barriers per consultation was capped at 4, although there may have been additional legal needs that were not able to be addressed due to time limitations.

^b^ Barriers were ranked in order of priority by legal consultants following each encounter, based on the amount of time spent discussing each barrier. A total of 3003 participants (51.7%) discussed 1 barrier; 1869 (32.2%), 2 barriers; 738 (12.7%), 3 barriers; and 200 (3.4%), 4 barriers.

### Patient Factors Associated With Legal Barriers

**Figure 2.**
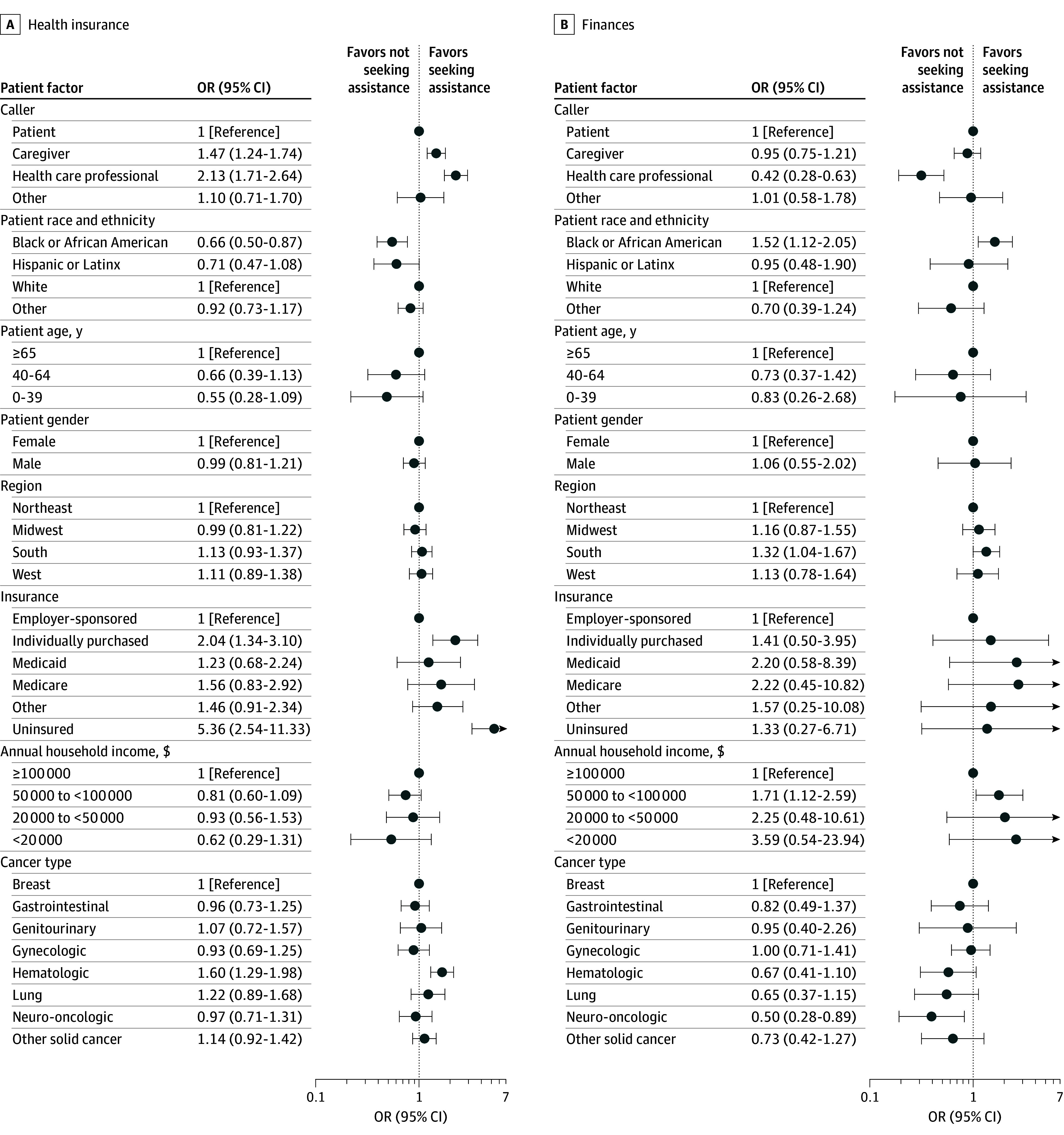
Patient Factors Associated With Legal Barriers Related to Health Insurance and Finances For each regression, the primary barrier was grouped into a binary outcome (eg, health insurance as the primary barrier vs all other issue areas as the primary barrier) and evaluated against all covariates. Multiple imputation was used to account for missing data. Individuals reporting nonbinary, transgender, or other for gender; international or US for territory; and high risk or seeking preventive health care services for cancer type were removed from the regression, as sample sizes were too small for analysis. The probability of each legal barrier by patient factor is given in eTable 3 in Supplement 1. OR indicates odds ratio.

**Figure 3.**
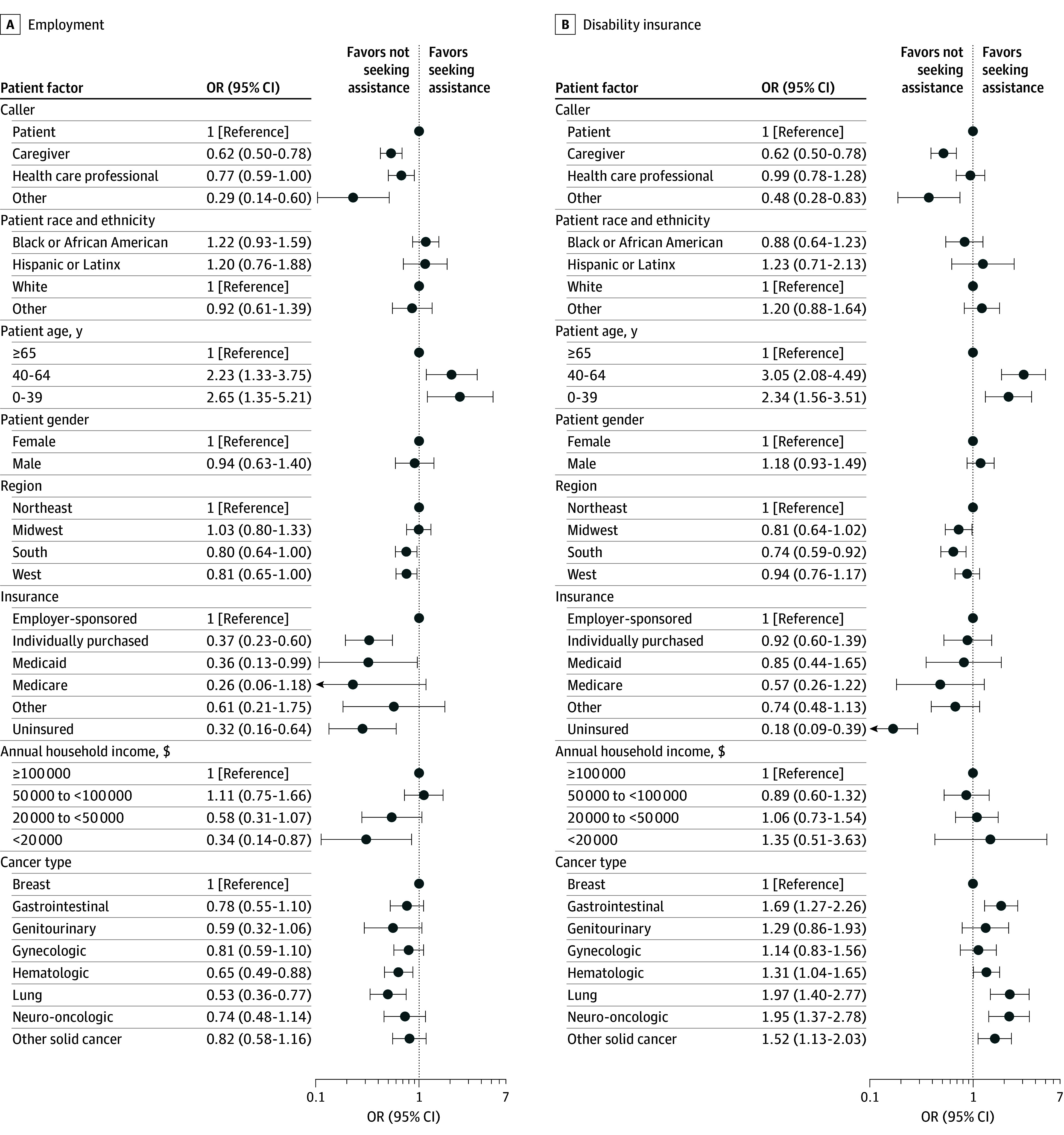
Patient Factors Associated With Legal Barriers Related to Employment and Disability Insurance For each regression, the primary barrier was grouped into a binary outcome (eg, health insurance as the primary barrier vs all other issue areas as the primary barrier) and evaluated against all covariates. Multiple imputation was used to account for missing data. Individuals reporting nonbinary, transgender, or other for gender; international or US for territory; and high risk or seeking preventive health care services for cancer type were removed from the regression, as sample sizes were too small for analysis. The probability of each legal barrier by patient factor is given in eTable 3 in Supplement 1. OR indicates odds ratio.

After adjusting for other covariates, individuals identifying as Black or African American had lower odds of calling with a primary legal barrier of health insurance compared with White individuals (OR, 0.66; 95% CI, 0.50-0.87) (Figure 2 and Figure 3). Caregivers (OR, 1.47; 95% CI, 1.24-1.74) and health care professionals (OR, 2.13; 95% CI, 1.71-2.64) had higher odds of calling on behalf of patients for health insurance issues compared with patients calling themselves. Uninsured individuals (OR, 5.36; 95% CI, 2.54-11.33) and those with individually purchased marketplace insurance (OR, 2.04; 95% CI, 1.34-3.10) had greater odds of seeking assistance for health insurance issues compared with individuals with employer-sponsored insurance.

Black or African American individuals had higher odds of calling for assistance with financial barriers (OR, 1.52; 95% CI, 1.12-2.05) compared with White individuals, as did individuals living in the South (OR, 1.32; 95% CI, 1.04-1.67) compared with the Northeast and households with annual income of $50 000 to less than $100 000 (OR, 1.71; 95% CI, 1.12-2.59) compared with over $100 000. Seeking assistance with employment was less common among those with individually purchased marketplace insurance (OR, 0.37; 95% CI, 0.23-0.60) or Medicaid (OR, 0.36; 95% CI, 0.13-0.99) and those who were uninsured (OR, 0.32; 95% CI, 0.16-0.64) than those with employer-sponsored insurance and among households with income below $20 000 (OR, 0.34; 95% CI, 0.14-0.87) compared with over $100 000. In addition, individuals aged 40 to 64 years (OR, 3.05; 95% CI, 2.08-4.49) and 0 to 39 years (OR, 2.34; 95% CI, 1.56-3.51) had higher odds of making calls for disability insurance compared with those aged 65 years or older, as did individuals with lung (OR, 1.97; 95% CI, 1.40-2.77), neurologic (OR, 1.95; 95% CI, 1.37-2.78), and gastrointestinal (OR, 1.69; 95% CI, 1.27-2.26) cancers compared with those with breast cancer. Probabilities of health insurance, financial, employment, and disability insurance barriers by individual patient factors are given in eTable 3 in Supplement 1.

## Discussion

In this nationwide analysis of a heterogeneous cohort of patients with cancer and their caregivers and health care professionals, we identified not only the most common legal barriers impacting patients but the extent to which the study population identified 2 or more barriers requiring legal navigation. These diverse barriers included health insurance, financial assistance, disability insurance, and employment status as well as less commonly recognized barriers such as estate planning, life insurance, and family law. The prevalence of these barriers varied with disease site and financial and sociodemographic factors. Altogether, this study found a demand for accessible legal navigation services and education for health care professionals to help address legal barriers to cancer care access. It highlights the potential for state- and federal-level policy efforts in building legal safeguards to enhance care.

Approximately 300 medical-legal partnerships addressing health-related legal needs exist today in the US, helping patients with utilities assistance, custody plan development, will execution, and public benefits enrollment.^26,27,28,29,30,31^ While some of these partnerships serve oncology patients, there remain limitations in who they serve, what issues they can address, and geographic scope—for example, they often exclude individuals earning more than 125% of the federal poverty level. Moreover, comprehensive data on the frequency of such legal barriers is lacking; to our knowledge, prior to this study, only a 2007 legal brief with 51 patients has explored this topic.^32^ This current, nationwide study of 5810 individuals thus fills a gap, revealing diverse legal barriers across all sociodemographic levels.

Half of the study cohort required assistance with 2 or more legal barriers, with many individuals seeking help beyond the financial and health insurance–related concerns commonly discussed in oncologic literature. Notably, 18.2% sought help with disability insurance and 17.6% with employment-related concerns, highlighting underrecognized areas of vulnerability. These findings contribute to a growing body of literature on these issues, connecting such barriers to a complex legal landscape that may hinder access to benefits.^17,33,34^

The economic impact of unmet legal needs is equally significant. Each of the 4 legal barriers identified—health insurance, financial matters, employment, and disability insurance—have implications for financial hardship. In all of these domains, legal assistance has been shown to have major cost-saving potential, helping patients recover millions of dollars in health care expenses, successfully overturning denied insurance claims, and relieving medical debt.^35,36,37^ As cancer is one of the diseases with greatest expenditure within the US, integrating screening and referral systems for legal needs into routine cancer care may not only enhance patient support but also serve as a sustainable strategy to reduce escalating health care costs in the US.^38,39^

We situated this study in the context of recent polices supporting the intersection of legal and health care needs. At the practice level, the Centers for Disease Control and Prevention’s Accelerating Health Equity, Advancing Through Discovery initiative seeks to expand funding to strengthen medical-legal partnership care models for wider dissemination.^40,41^ Awareness of and training for legal barriers have been shown to influence practice patterns and increase likelihood of screening for social determinants of health, including housing, public benefits, and other important sociolegal needs.^42^ Development of National Comprehensive Cancer Network standards for screening for legal barriers can support these efforts further, equipping health care professionals to go beyond identifying financial assistance needs and assess underlying legal contributors to financial hardship, such as employment issues, access to benefits, and insurance denials. At the state and federal levels, the Centers for Medicare & Medicaid Services (CMS) introduced Section 1115 waivers to help states address health-related social needs, while the Health Resources and Services Administration has recognized legal aid as a key enabling service for primary care access, facilitating the use of Section 330 funds for legal navigation.^43,44^ CMS also established Principal Illness Navigation services, allowing reimbursement for coordinating care of patients with cancer.^45,46^ Further legal safeguards and regulatory standards are needed to ensure continued access to care. However, more recent policy changes in the 2025 Federal Budget Reconciliation Bill aim to restrict the federal budget, in part by increasing barriers to enrollment in public benefits like Medicaid.^47^ It is anticipated that state and federal offices may not only offer fewer benefits but also increase administrative burden on patients filing for eligibility, thereby potentially increasing demand for legal navigation services.

### Limitations

This study has limitations. First, while Triage Cancer reaches over 1 million individuals annually across 50 states, exposure to the organization may vary. As referrals often come from health care professionals and patient organizations, use may be biased toward individuals more integrated into health care networks and/or better informed to seek out a resource such as Triage Cancer. Individuals who may require legal navigation but are unable to receive services were not captured in this study, potentially underestimating the true prevalence of legal barriers. Second, some patient information was provided by proxies (caregivers and health care workers), introducing information bias. Third, as some calls were conducted by caregivers, we were unable to differentiate whether legal barriers were in reference to caregivers or patients, an important but separate aspect of care. Fourth, although multiple imputation was used to address incomplete data, this model assumed that data were missing at random, while in reality, missing data may be biased toward subsets of the cohort. Fifth, to protect privacy, patient identifiers were not recorded. As a result, a small proportion of our demographic, financial, and disease-site data may have been duplicated if patients had multiple calls to discuss different legal issues.

## Conclusions

In this cohort study evaluating patients with cancer seeking legal navigation services to address barriers to care, the most common primary legal barriers were related to health insurance, financial concerns, employment protections, and disability insurance. The findings not only demonstrate the number of legal barriers faced by various sociodemographic groups but also highlight vulnerable populations with greater need of legal navigation, informing strategies for education, practice, and policy. Health care teams could leverage existing systems and partnerships to provide additional training around legal topics to patient-facing staff, and research teams could further explore the impact of legal barriers faced by patients with cancer and develop interventions that evaluate the effectiveness of legal navigation in reducing financial distress and improving outcomes and quality of life for both patients and caregivers. Additionally, scholars at the intersection of health care and policy could advocate for laws and policies that close gaps in protection for the most vulnerable populations, including but not limited to expanding Medicaid in states, reducing wait times for Social Security Disability Insurance, and providing paid family and medical leave.

## Notes

Supplement 1. **eTable 1.** Classification of Legal Barriers **eFigure.** Map of US by Frequency of Calls **eTable 2.** Demographics of Cohort by Primary Legal Barrier **eTable 3.** Probability of Legal Barriers by Patient Factors

Supplement 2.
Data Sharing Statement
