# Supplementary material for: Nationwide Analysis of Legal Barriers to Cancer Care
Source: JAMA Netw Open. 2025 Jul 31;8(7):e2524201. doi: 10.1001/jamanetworkopen.2025.24201 (PMC12314725; doi:10.1001/jamanetworkopen.2025.24201)
Supplement: Supplement 1. — eTable 1. Classification of Legal Barriers eFigure. Map of US by Frequency of Calls eTable 2. Demographics of Cohort by Primary Legal Barrier eTable 3. Probability of Legal Barriers by Patient Factors [file jamanetwopen-e2524201-s001.pdf]

Supplementary Online Content

Chen KY, Blackford AL, Bryant MF, et al. Nationwide analysis of legal barriers to cancer care. *JAMA Netw Open*. 2025;8(7):e2524201.
doi:10.1001/jamanetworkopen.2025.24201

eTable 1. Classification of Legal Barriers

eFigure. Map of US by Frequency of Calls

eTable 2. Demographics of Cohort by Primary Legal Barrier

eTable 3. Probability of Legal Barriers by Patient Factors

This supplementary material has been provided by the authors to give readers additional information about their work.

© 2025 Chen KY et al. *JAMA Network Open*.

eTable 1. Categories of Legal Barriers

Category	Specific Legal Concern
Health Insurance	Health Insurance Claim Denial Appeals<br>Health Insurance Navigation<br>Medicaid<br>Medicare<br>Understanding Options
Financial	Financial Assistance<br>Housing<br>Other Financial Issue
Employment	Caregiver Rights<br>Getting Fired<br>Job Search<br>Returning to Work<br>Taking Time Off<br>Unemployment Benefits<br>Working Through Treatment<br>Other Employment Issue
Disability Insurance	Applying for Disability Insurance<br>Disability Insurance Claim Denial Appeals<br>Transitioning Off Disability Insurance<br>Other Disability Insurance Issues
Other	COVID-19<br>Education<br>Estate Planning, Wills, Advanced Directives<br>Family Law<br>Immigration<br>Life Insurance<br>Other Insurance Issue<br>Other

© 2025 Chen KY et al. *JAMA Network Open*.

eFigure. **Map of US by Frequency of Calls[a]**

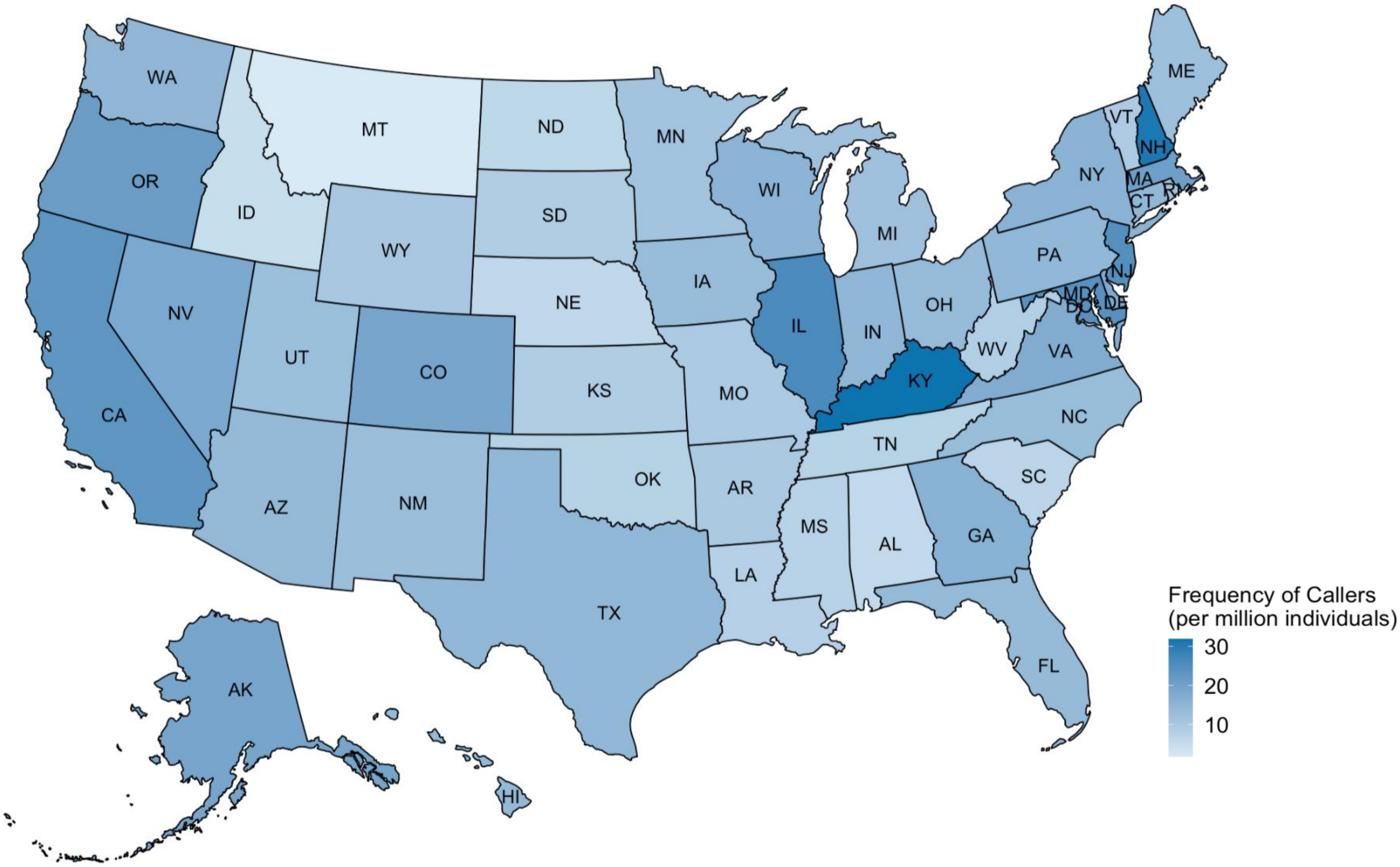

[a] Darker hues represent increased number of calls while lighter hues represent decreased number of calls. Color scale was normalized by state population.

© 2025 Chen KY et al. *JAMA Network Open*.

eTable 2. Demographics of Cohort by Primary Legal Barrier[a]

Variable	Classification	All (N = 5810)	Health (N = 1648)	Financial (N = 1194)	Employment (N = 1095)	Disability (N = 1082)	Other (N = 791)
Types of Callers	Individual Diagnosed with Cancer	3883 (66.8)	960 (58.3)	832 (69.7)	847 (77.4)	783 (72.4)	461 (58.3)
	Caregiver	1091 (18.8)	363 (22.0)	122 (17.4)	136 (12.4)	154 (14.2)	201 (25.4)
	Social Worker	400 (6.9)	148 (9.0)	38 (3.2)	76 (6.9)	100 (9.2)	38 (4.8)
	Community Health Worker/Patient Advocate	99 (1.7)	36 (2.2)	19 (1.6)	7 (0.6)	14 (1.3)	23 (2.9)
	Nurse	72 (1.2)	42 (2.5)	11 (0.9)	10 (0.9)	4 (0.4)	5 (0.6)
	Financial Navigator	26 (0.4)	16 (1.0)	2 (0.2)	1 (0.1)	4 (0.4)	3 (0.4)
	Other[b]	229 (3.9)	80 (4.9)	53 (4.4)	16 (1.5)	23 (2.1)	57 (7.2)
	Unknown	10 (0.2)	3 (0.3)	2 (0.2)	2 (0.2)	0 (0.0)	3 (0.4)
Age of Patient at Time of Call (years)	0-39	968 (16.7)	259 (15.7)	181 (15.2)	208 (19.0)	180 (16.6)	140 (17.7)
	40-64	3293 (56.7)	878 (53.3)	575 (48.2)	750 (68.5)	758 (70.1)	332 (42.0)
	65+	807 (13.9)	328 (19.9)	205 (17.2)	55 (5.0)	61 (5.6)	158 (20.0)
	Unknown/Prefer not to share	742 (12.8)	183 (11.1)	233 (19.5)	82 (7.5)	83 (7.7)	161 (20.4)
Gender of Patient	Female	3710 (63.9)	997 (60.5)	761 (63.7)	787 (71.9)	675 (62.4)	490 (61.9)
	Male	1697 (29.2)	538 (32.6)	326 (27.3)	253 (23.1)	361 (33.4)	219 (27.7)
	Other[c]	32 (0.6)	10 (0.6)	4 (0.3)	4 (0.4)	6 (0.6)	8 (1.0)
	Unknown/Prefer not to share	371 (6.4)	103 (6.3)	103 (8.6)	51 (4.7)	40 (3.7)	74 (9.4)
Race/Ethnicity	White	2840 (48.9)	897 (54.4)	440 (36.9)	593 (54.2)	583 (53.9)	327 (41.3)
	Hispanic or Latinx	598 (10.3)	161 (9.8)	122 (10.2)	121 (11.1)	121 (11.2)	73 (9.2)
	Black or African American	597 (10.3)	126 (7.6)	192 (16.1)	113 (10.3)	99 (9.1)	67 (8.5)
	Asian or Pacific Islander	277 (4.8)	72 (4.4)	27 (2.3)	71 (6.5)	69 (6.4)	38 (4.8)
	Other race, ethnicity, or origin[d]	129 (2.2)	30 (1.8)	29 (2.4)	17 (1.6)	30 (2.8)	23 (2.9)

© 2025 Chen KY et al. *JAMA Network Open*.

	Middle Eastern of North African	51 (0.9)	18 (1.1)	14 (1.2)	7 (0.6)	7 (0.6)	5 (0.6)
	American Indian or Alaska Native	17 (0.3)	5 (0.3)	5 (0.4)	3 (0.3)	2 (0.2)	2 (0.3)
	Unknown/Prefer not to share	1301 (22.4)	339 (20.6)	365 (30.6)	170 (15.5)	171 (15.8)	256 (32.4)
Region	South	1786 (30.7)	540 (32.8)	398 (33.3)	309 (28.2)	299 (27.6)	240 (30.3)
	West	1482 (25.5)	441 (26.8)	249 (20.9)	287 (26.2)	325 (30.0)	190 (24.0)
	Midwest	1011 (17.4)	288 (17.5)	180 (15.1)	220 (20.1)	191 (17.7)	132 (16.7)
	Northeast	997 (17.2)	272 (16.5)	161 (13.5)	227 (20.7)	218 (20.1)	119 (15.0)
	International	82 (1.4)	15 (0.9)	50 (4.2)	1 (0.1)	0 (0.0)	16 (2.0)
	US Territory	4 (0.1)	1 (0.1)	1 (0.1)	0 (0.0)	1 (0.1)	1 (0.1)
	Unknown	448 (7.7)	91 (5.5)	155 (13.0)	51 (4.7)	48 (4.4)	93 (11.8)
Primary Language Other than English		167 (3.2)	52 (3.2)	40 (3.4)	30 (2.7)	23 (2.1)	22 (2.8)
Primary Health Insurance	Employer-Sponsored	1977 (34.0)	472 (28.6)	195 (16.3)	674 (61.6)	455 (42.1)	181 (22.9)
	Medicare	862 (14.8)	324 (19.7)	238 (19.9)	50 (4.6)	93 (8.6)	157 (19.8)
	Medicaid	745 (12.8)	159 (9.6)	232 (19.4)	74 (6.8)	179 (16.5)	101 (12.8)
	Individually-purchased	585 (10.1)	217 (13.2)	96 (8.0)	76 (6.9)	138 (12.8)	58 (7.3)
	Uninsured	330 (5.7)	183 (11.1)	78 (6.5)	29 (2.6)	14 (1.3)	26 (3.3)
	Other (other, military, or veteran)	267 (4.6)	83 (5.0)	41 (3.4)	40 (3.7)	53 (4.9)	34 (4.3)
	Unknown	1044 (18.0)	210 (12.7)	314 (26.3)	152 (13.9)	150 (13.9)	234 (29.6)
Employment Status	Employed	2313 (39.8)	531 (32.2)	318 (26.6)	763 (69.7)	516 (47.7)	185 (23.4)
	Unemployed – Unable to Work	518 (8.9)	168 (10.2)	121 (10.1)	30 (2.7)	120 (11.1)	79 (10.0)
	Unemployed – Looking for Work	505 (8.7)	126 (7.6)	143 (12.0)	55 (5.0)	115 (10.6)	66 (8.3)
	Retired	340 (5.9)	173 (10.5)	74 (6.2)	5 (0.5)	31 (2.9)	57 (7.2)
	Leave	33 (0.6)	13 (0.8)	4 (0.3)	5 (0.5)	8 (0.7)	3 (0.4)
	NA[e]	82 (1.4)	30 (1.8)	12 (1.0)	8 (0.7)	14 (1.3)	18 (2.3)
	Unknown	1935 (33.3)	607 (36.8)	522 (43.7)	229 (20.9)	278 (25.7)	383 (48.4)

© 2025 Chen KY et al. *JAMA Network Open*.

Household Income	Below $13,000	907 (15.6)	197 (12.0)	129 (10.8)	87 (7.9)	206 (19.0)	126 (15.9)
	$13,000-$20,000	471 (8.1)	152 (9.2)	236 (19.8)	45 (4.1)	72 (6.7)	73 (9.2)
	$20,000-$50,000	1155 (19.9)	355 (21.5)	80 (6.7)	208 (19.0)	219 (20.2)	137 (17.3)
	$50,000-$100,000	993 (17.1)	272 (16.5)	328 (27.5)	306 (27.9)	202 (18.7)	96 (12.1)
	$100,000+	581 (10.0)	166 (10.1)	33 (2.8)	188 (17.2)	139 (12.8)	55 (7.0)
	Unknown/Prefer not to share	1703 (29.3)	506 (30.7)	388 (32.5)	261 (23.8)	244 (22.6)	304 (38.4)
Household Size	1	1648 (28.4)	461 (28)	361 (30.2)	310 (28.3)	312 (28.8)	204 (25.8)
	2	1597 (27.5)	533 (32.3)	261 (21.9)	314 (28.7)	314 (29.0)	175 (22.1)
	3	668 (11.5)	165 (10.0)	123 (10.3)	148 (13.5)	149 (13.8)	83 (10.5)
	4+	778 (13.4)	177 (10.7)	139 (11.6)	187 (17.1)	167 (15.4)	108 (13.7)
	Unknown	1119 (19.3)	312 (18.9)	310 (26.0)	136 (12.4)	140 (12.9)	221 (27.9)
Cancer	Breast	1618 (27.8)	392 (23.8)	341 (28.6)	415 (37.9)	270 (25.0)	200 (25.3)
	Hematologic	1134 (19.5)	394 (23.9)	219 (18.3)	188 (17.2)	203 (18.8)	130 (16.4)
	Gastrointestinal	572 (9.8)	152 (9.2)	119 (10.0)	103 (9.4)	132 (12.2)	66 (8.3)
	Gynecologic	358 (6.2)	89 (5.4)	75 (6.3)	77 (7.0)	63 (5.8)	54 (6.8)
	Lung	355 (6.1)	113 (6.9)	66 (5.5)	44 (4.0)	86 (7.9)	46 (5.8)
	Neuro-oncologic	319 (5.5)	86 (5.2)	37 (3.1)	60 (5.5)	78 (7.2)	58 (7.3)
	Genitourinary	224 (3.9)	66 (4.0)	61 (5.1)	27 (2.5)	37 (3.4)	33 (4.2)
	High risk/seeking preventative services	33 (0.6)	20 (1.2)	2 (0.2)	2 (0.2)	5 (0.5)	4 (0.5)
	Other Solid Cancer	689 (11.9)	197 (12.0)	135 (11.3)	129 (11.8)	143 (13.2)	85 (10.7)
	Unknown	508 (8.7)	139 (8.4)	139 (11.6)	50 (4.6)	65 (6.0)	115 (14.5)
Stage of Treatment	Pre Treatment	350 (6.0)	117 (7.1)	86 (7.2)	65 (5.9)	49 (4.5)	33 (4.2)
	In Treatment	2808 (48.3)	681 (41.3)	610 (51.1)	600 (54.8)	591 (54.6)	326 (41.2)
	Post Treatment	717 (12.3)	157 (9.5)	135 (11.3)	182 (16.6)	141 (13.0)	102 (12.9)
	Unknown	1935 (33.3)	693 (42.1)	363 (30.4)	248 (22.6)	301 (27.8)	330 (41.7)

[a] Information was self-reported by participants in a survey prior to consultation. Information was reported for the patient experiencing legal barriers, not the participant serving as their proxy.
[b] Includes high risk individuals seeking preventive services and other callers.
[c] Includes non-binary-non-conforming, transgender, gender not listed.

© 2025 Chen KY et al. *JAMA Network Open*.

[d] Participants self-reported as “other race, ethnicity, origin.” This category does not include any groupings made by the research team after data collection.
[e] Includes children, students, stay-at-home parents.

© 2025 Chen KY et al. *JAMA Network Open*.

eTable 3. Probability of Legal Barriers by Patient Factor

Patient factor	Probability of Legal Barrier			
	Health Insurance	Financial	Employment	Disability Insurance
Caller				
Patient	0.25	0.20	0.22	0.20
Caregiver	0.34	0.19	0.13	0.15
Health Care Worker	0.41	0.11	0.15	0.20
Other	0.29	0.22	0.06	0.11
Race and Ethnicity				
White	0.30	0.18	0.19	0.19
Black or African American	0.23	0.29	0.18	0.16
Hispanic or Latinx	0.26	0.19	0.19	0.21
Other	0.28	0.13	0.19	0.23
Age, y				
≥65	0.39	0.27	0.07	0.07
40-64	0.27	0.17	0.21	0.22
0-39	0.25	0.19	0.22	0.18
Gender				
Female	0.27	0.20	0.21	0.18
Male	0.32	0.18	0.15	0.21
Region				
Northeast	0.27	0.17	0.22	0.21
Midwest	0.27	0.19	0.22	0.18
South	0.30	0.22	0.17	0.17
West	0.29	0.18	0.18	0.21

© 2025 Chen KY et al. *JAMA Network Open*.

Insurance				
Employee-sponsored	0.22	0.11	0.32	0.22
Individual	0.35	0.19	0.12	0.22
Medicaid	0.22	0.30	0.10	0.23
Medicare	0.34	0.28	0.06	0.11
Other	0.29	0.19	0.20	0.19
Uninsured	0.56	0.20	0.09	0.06
Household Income, $				
≥100 000	0.29	0.07	0.31	0.21
50 000 to 100 000	0.27	0.13	0.32	0.18
20 000 to 50 000	0.33	0.19	0.15	0.18
<20 000	0.26	0.28	0.08	0.21
Cancer Type				
Breast	0.24	0.22	0.25	0.16
Gastrointestinal	0.26	0.20	0.17	0.23
Genitourinary	0.30	0.24	0.12	0.17
Gynecologic	0.25	0.21	0.21	0.17
Hematologic	0.35	0.17	0.16	0.18
Lung	0.32	0.17	0.12	0.24
Neurologic	0.27	0.12	0.18	0.24
Other	0.30	0.18	0.18	0.20

© 2025 Chen KY et al. *JAMA Network Open*.
