# Supplementary material for: Nationwide Analysis of Legal Barriers to Cancer Care
Source: JAMA Netw Open. 2025 Jul 31;8(7):e2524201. doi: 10.1001/jamanetworkopen.2025.24201 (PMC12314725; doi:10.1001/jamanetworkopen.2025.24201)
Supplement: Supplement 2. — Data Sharing Statement [file jamanetwopen-e2524201-s002.pdf]

Data Sharing Statement

Chen. Nationwide Analysis of Legal Barriers Impacting Cancer Care. *JAMA Netw Open*. Published July 31, 2025. doi:10.1001/jamanetworkopen.2025.24201

Data
Data available: Yes
Data types: Deidentified participant data
How to access data: We will provide these as required
When available: With publication

Supporting Documents
Document types: Statistical/analytic code
How to access documents: Will provide these as needed
When available: With publication

Additional Information
Who can access the data: None
Types of analyses: As noted
Mechanisms of data availability: Email
Any additional restrictions: None
